# Antimicrobial susceptibility profile of *Klebsiella pneumoniae* isolated from some dairy products in Libya as a foodborne pathogen

**DOI:** 10.14202/vetworld.2024.1168-1176

**Published:** 2024-05-22

**Authors:** Salah M. Azwai, Aml F. Lawila, Hanan L. Eshamah, Jihan A. Sherif, Samira A. Farag, Hesham T. Naas, Aboubaker M. Garbaj, Allaaeddin A. El Salabi, Fatim T. Gammoudi, Ibrahim M. Eldaghayes

**Affiliations:** 1 Department of Microbiology and Parasitology, Faculty of Veterinary Medicine, University of Tripoli, Tripoli, Libya; 2 Food and Drug Control Center, Tripoli, Libya; 3 Department of Food Hygiene and Control, Faculty of Veterinary Medicine, University of Tripoli, Tripoli, Libya; 4 Department of Public Health, Faculty of Medical Technology, University of Tripoli, Tripoli, Libya

**Keywords:** antibiotic sensitivity, dairy products, foodborne, *Klebsiella pneumoniae*, Libya

## Abstract

**Background and Aim:**

*Klebsiella pneumoniae* is one of the most common causes of clinical and asymptomatic mastitis in dairy cattle, as well as in milk and dairy products that affect milk quality. Mastitis caused by *K. pneumoniae* is even more serious due to its poor response to antibiotic therapy. The aim of this study was to detect and identify the presence of *K. pneumoniae* in milk and dairy products produced in Libya.

**Materials and Methods:**

A total of 234 samples were randomly collected from various locations in Libya. Samples were examined for the presence of *K. pneumoniae* using conventional cultural techniques, including cultivation in violet red bile agar plus 4-methylumbelliferyl-ß-D-glucuronide broth and CHROM agar, followed by polymerase chain reaction identification and partial sequencing of 16S rRNA.

**Results:**

Of the 234 samples of milk and dairy products collected, 16 (6.8%) isolates revealed mucoid colonies on agar media that were phenotypically suggested to be *K. pneumoniae*. Identification of isolates was confirmed using molecular techniques (16S rRNA). Among the examined samples, *K. pneumoniae* was recovered from camel’s milk, raw cow’s milk, raw fermented milk, Maasora cheese, Ricotta cheese, soft cheese, full cream milk powder, milk powder infant formula, cereal baby food, and growing-up formula. Antibiotic susceptibility testing was performed on 12 of the 16 *K. pneumoniae* isolates, and the results showed that *K. pneumoniae* isolates were resistant to more than eight antibiotics; interestingly, two isolates showed metallo-beta-lactamase (MBL) production.

**Conclusion:**

*K. pneumoniae* is considered a risk to human health because many of these products do not comply with the microbiological criteria of international and/or Libyan standards. This study emphasized the relationship between *K. pneumoniae* and raw milk, cheese, milk powder, and infant milk retailed in Libya. There is a need to take the necessary measures to ensure effective hygiene practices during production in dairy factories, handling, and distribution on the market, in particular at a small local production scale.

## Introduction

*Klebsiella pneumoniae* is a Gram-negative pathogen. It is an important member of the Enterobacteriaceae family that can infect humans through contaminated poultry, beef, fish, and dairy products [1]. *K. pneumoniae* has a mucoid phenotype on agar medium, which is imparted by the polysaccharide capsule attached to the outer membrane of the cell, resulting in lactose fermentation [2]. *K. pneumoniae* has emerged as a clinically and epidemiologically important human pathogen due to its ability to survive in different environments such as surfaces, human skin, and respiratory and urinary tract. *K. pneumoniae* is easily transmitted between patients through surgery and has become one of the most common causes of outbreaks in intensive care units [3]. *K. pneumoniae* is a common bacterial pathogen that accounts for a high proportion of nosocomial infections in pediatric patients and can cause life-threatening invasive infections in young people [4].

Sources of *Klebsiella* species on dairy farms include organic bedding materials, such as wood by-products and fecal excrement from cattle, which contain a wide variety of *K. pneumoniae* strains in their dairy products [5]. To avoid this problem, microbial agents are added to dairy cow feed to inhibit pathogenic bacteria [6].

Mastitis is one of the most serious diseases in dairy herds, despite the widespread use of management programs such as teat immersion, dry cow treatment, and weaning of infected animals. Mastitis caused by *K. pneumoniae* is particularly severe compared with that caused by *Escherichia coli* because of its poor response to antibiotic therapy and rapid progression to toxic shock and death [7]. In addition, this bacterium can cause severe clinical symptoms and significant economic losses [8]. *K. pneumoniae* ST1224 and *K. pneumoniae* ST48 were isolated from neonates and chicken meat, respectively, in Western Algeria, one of the Middle East regions, indicating that these *Klebsiella* isolates are not host-specific and could be easily transmitted to humans from food animals and their products [9].

*K. pneumoniae* is an opportunistic pathogen for individuals with weakened immunity because it can cause various infections, including pneumonia, urinary tract infection, bacteremia, and meningitis. In contrast to “classical” *K. pneumoniae*, “hypervirulent” *K. pneumoniae* with hypermucoviscosity has emerged as a clinically important pathogen over the last two decades. It causes highly invasive infections, such as liver abscesses, in healthy and immunocompromised individuals [10]. Antibacterial resistance to certain infectious pathogens has become a serious public health concern. One study documented that food-producing animals are a possible source or reservoir for the spread of resistant bacterial strains or antibiotic-resistant genes to humans [1].

*Pseudomonas aeruginosa*, *Acinetobacter baumannii*, and *K. pneumoniae* are well-known nosocomial pathogens. Recently, there has been a worldwide surge in multidrug resistance (MDR) and pandrug-resistant counterparts on the World Health Organization list of priority (serious) pathogens of antibiotic resistance. Surveillance studies have reported that resistance of *K. pneumoniae* to third-generation cephalosporins peaked at 15%–20%, and ciprofloxacin resistance peaked at 10%–50%) [11].

Interestingly, the population of *K. pneumoniae* contains numerous extraordinary genes comprising unbiased phylogenetic lineages or “clones” that fluctuate from one another. The majority of MDR sanatorium outbreaks are due to a small subset of *K. pneumoniae* clones with an excessive incidence of received antimicrobial resistance (AMR) genes, whereas the bulk of community-received invasive infections are due to “hypervirulent” clones that do not often harbor received AMR genes, but have an excessive incidence of key virulence loci [10]. Empirical use of antibiotics and persistent exposure to various antimicrobials have led to an increased prevalence of MDR *K. pneumoniae* [4].

Resistance to the majority of antimicrobial classes and the lack of new antimicrobial agents against Gram-negative bacteria can lead to the re-use of old antibiotics, in particular polymyxins such as colistin. Colistin is currently used as a last-resort antimicrobial agent for the treatment of infections caused by Gram-negative MDR bacteria. It binds to the negatively charged lipopolysaccharide of the outer membrane of Gram-negative bacteria, disrupting the membrane, causing the cytoplasmic material to fade away and, eventually, cell death.

The indiscriminate use of antimicrobials has led to the development of MDR Gram-negative bacteria in raw milk, particularly *E. coli* O157:H7, *K. pneumoniae*, *Aeromonas hydrophilia*, and *Proteus mirabilis* [9].

Over the past few years, Gram-negative bacteria have shown a significant increase in resistance against β-lactam antibiotics due to various plasmid-mediated extended spectrum β-lactamases (ESBL) genes found in Enterobacteriaceae, especially in *E. coli* and *K. pneumoniae*. The emergence of resistant *K. pneumoniae* due to the misuse of antibiotics in ranches and veterinary facilities is a serious public and livestock health problem. Ultimately, it can be transmitted to humans through different environmental niches [1]. The emergence of MDR bacterial pathogens from different origins, including humans, birds, cattle, and fish, necessitates the need for new potent and safe antimicrobial agents [12].

ESBL hydrolyzes various β-lactam antibiotics and confers resistance to penicillin and third- and fourth-generation cephalosporins. The genes encoding these enzymes are common in chromosomes and plasmids of Enterobacteriaceae species. Therefore, Enterobacteriaceae, especially *E. coli* and *Klebsiella* spp. Various groups of antibiotics are used in livestock management at both therapeutic and subtherapeutic levels. β-lactam antibiotics are widely used in veterinary medicine because of their high specificity, complete selective toxicity, and strong killing effect. Therefore, abuse of these antibiotics in veterinary medicine increased the emergence and spread of genetic determinants, especially in *E. coli* and *K. pneumoniae* [13].

ESBL-producing *K. pneumoniae* has been isolated from food handlers who consumed unpasteurized milk and raw meat [14]. Recently, *K. pneumoniae* has been detected in human samples, again emphasizing the concept of transfer between animal/animal products, including milk, from/to humans and animals during the last decade. MDR *K. pneumoniae* isolates have also been widely detected in milk samples [9]. *K. pneumoniae* is considered to be a major transporter of resistance genes from environmental sources to clinically important bacteria, and some isolates can carry acquired AMR genes or plasmids to move between environments, humans, and animals [12].

*E. coli*, *Cronobacter* spp., and *Salmonella* *enterica* have previously been reported by Garbaj *et al*. [15], Garbaj *et al*. [16] and Garbaj *et al*. [17] as foodborne pathogens associated with dairy products in Libya. Data on the microbiological quality of Libyan dairy products related to *K. pneumoniae* are lacking. Previously, much information has been recorded only on mastitic cases; although this is not a common cause of food poisoning, it is known to be a causative agent of mastitis. As a result of the lack of standard manufacturing guidelines, there has been a lack of hygienic practices during the manufacture of dairy products, in particular in locally produced milk products. All these factors are considered to be the greatest public health risk in Libya.

Therefore, this study aimed to investigate the presence of *K. pneumoniae* in milk and dairy products sold in Libya and to determine its antibiotic sensitivity profile.

## Materials and Methods

### Ethical approval

Dairy samples used in the research were collected from various retail dairy markets from different locations in Libya.

### Study period and location

The study was conducted from January 2019 to December 2020. Samples of milk and dairy products were collected from different Libyan cities (Tripoli, Sabha, Tobruk, and Regdalin).

### Sampling

A total of 234 samples of milk and dairy products (Table-1) were randomly collected from different locations in Libya.

**Table 1:** Total number of samples produced *Klebsiella pneumoniae* on agar media.

Type of samples	Total number of samples	Number of positive samples
Raw cow’s milk	46	3 (6.5%)
Raw she camel’s milk	5	1 (20%)
Raw fermented milk	28	1 (3.5%)
UHT milk	8	0
Yoghurt	5	0
Maasora cheese	21	4 (19%)
Ricotta cheese	13	1 (7.6%)
Imported soft cheese	6	1 (16.6%)
Ice cream	6	0
Full cream milk powder	10	2 (20%)
Skimmed milk powder	6	0
Powder infant formula	36	1 (2.7%)
Growing up formula	18	1 (5.5%)
Ready to feed baby milk	10	0
Cereal baby food	16	1 (6.25%)
Total	234	16 (6.8%)

### Identification and isolation of *K. pneumoniae*

Isolation of *K. pneumoniae* was performed according to the reference method described by Davis *et al*. [18] for the detection of *K. pneumoniae* in dairy products. In brief, 25 g/mL from each sample was aseptically transferred into a sterile polyethylene stomacher bag and blended with 225 mL of buffer peptone water (Park Scientific, M 0063, Northampton Limited, UK), homogenized in a stomacher (Stomacher 400, Seaward Medicals, UK) at 1,000× *g* for 1 min, then incubated at 37°C for 18 ± 2 h. Next, 10 mL from the above-incubated samples was added to enrichment broth (violet red bile agar plus 4-methylumbelliferyl-ß-D-glucuronide) and incubated overnight at 44°C. Only 0.1 mL of the selectively enriched broth was streaked onto CHROM agar (Hardy Diagnostics, Santa Maria, California, USA), and the inoculated plates were incubated at 37°C for 24 h. Presumptive colonies were selected and maintained for further investigation [18].

### Identification of *K. pneumoniae* using polymerase chain reaction (PCR) and partial sequencing of 16S rDNA

Colonies cultivated on CHROM agar were selected and purified several times. DNA extraction from *K. pneumoniae* isolates was performed as previously described by Azwai *et al*. [19]. Partial 16S rDNA was amplified using universal oligonucleotide primers S-D Bact-0341-b-S-17 and S-D-Bact-0785-a- A-21. The amplified 16S rDNA PCR fragment (464 bp) was excised from the gel, and DNA was extracted from the gel using a GF-1 AmbiClean kit (Cat. # GF-GC-100, Vivantis, Malaysia) as reported by Azwai *et al*. [19]. The purified 16S rDNA amplicons were sequenced in IZSLER-Istituto Zooprofilattico Sperimentale della Lombardia e dell’Emilia Romagna, Brescia, Italy. Basic Local Alignment Search Tool search was performed on consensus reached by both NCBI (http://www.ncbi.nlm.nih.gov/pubmed) and 16S bacterial cultures Blast Server for the identification of prokaryotes (http://bioinfo.unice.fr/blast/).

### Antimicrobial susceptibility profile

#### Selected antibiotic disks

*K. pneumoniae* isolates confirmed by PCR were subsequently tested against a variety of 32 antibiotics using the disc-diffusion method described in the Clinical and Laboratory Standards Institute guidelines as described by Davis *et al*. [18]. Antibiotics (Oxoid, England) used included gentamycin (Bioanalyse 10 μg), streptomycin (10 μg), amoxicillin (10 μg), bacitracin (10 μg), oxytetracycline (30 μg), doxycycline (30 μg), penicillin G (10 μg), erythromycin (15 μg), amoxicillin/clavulanic acid (30 μg), ampicillin (10 μg), methicillin (Bioanalyse 5 μg), kanamycin (30 μg), lincomycin (10 μg), tobramycin (10 μg), levofloxacin (5 μg), clindamycin (2 μg), cefotaxime (30 μg), ciprofloxacin (5 μg), cloxacillin (5 μg), nitrofurantoin (300 μg), nitrofurantoin (30 μg), tetracycline (30 μg), nitrofurantoin (25 μg), piperacillin/tazobactam (100/10 μg), cephoxitin (30 μg), cefoperazone/sulbactam (75/30 μg), cefotaxime (30 μg), cephoxitin (30 μg), ceftazidime (30 μg), cefotaxime (30 μg), imipenem (10 μg), ertapenem (10 μg), and azobactam (30 μg).

#### Antibiogram assay

A single colony of the tested organism was picked up using a disposable loop, emulsified into 5 mL Brain Heart Infusion Broth (Liofilchem, Italy), and incubated overnight at 37°C to reach McFarland’s standard turbidity of 0.5. A cotton swab was used to spread the suspension on the surface of Mueller-Hinton agar (Oxoid, England). Suitable test antibiotic disks were placed on the agar surface, and the plates were incubated at 37°C for 24 h. The diameter of the inhibition zone was measured in millimeters and recorded.

## Results

Out of 234 samples of milk and dairy products, only 16 (6.4%) of the isolates revealed mucoid colonies on agar media that were phenotypically suggested to be *K. pneumoniae* (Table-1).

The isolate identification was confirmed by molecular techniques (16S rRNA), which identified 16 (6.4%) mucoid isolates as *K. pneumoniae* (Table-2). *K. pneumoniae* was recovered from camel’s milk, raw cow’s milk, and fermented milk as 1 (20%), 3 (6.5%), and 1 (3.5%), respectively, whereas no *K. pneumoniae* was detected in the ultra-high temperature (UHT) milk samples. *K. pneumoniae* was detected in 4 (19%), 1 (7.6%), and 1 (16.6%) cheese samples of Maasora cheese, Ricotta cheese, and imported soft cheese samples, respectively.

**Table 2:** Bacterial identification of 12 isolates confirmed to be *K. pneumoniae* by using the polymerase chain reaction-16S rDNA technique.

S. No.	Blast NCBI 16S rDNA	Nucleotide identity (%)	Type of samples
1	*K. pneumoniae*	99	Raw cow’s milk
2	*K. pneumoniae*	99	Raw cow’s milk
3	*K. pneumoniae*	99	Raw cow’s milk
4	*K. pneumoniae*	99	She-camel’s milk
5	*K. pneumoniae*	98	Raw fermented milk
6	*K. pneumoniae*	99	Ricotta cheese
7	*K. pneumoniae*	99	Maasora cheese
8	*K. pneumoniae*	99	Maasora cheese
9	*K. pneumoniae*	99	Maasora cheese
10	*K. pneumoniae*	100	Maasora cheese
11	*K. pneumoniae*	99	Imported soft cheese
12	*K. pneumoniae*	100	Full cream milk powder
13	*K. pneumoniae*	99	Full cream milk powder
14	*K. pneumoniae*	100	Infant formula
15	*K. pneumoniae*	99	Fortified milk powder
16	*K. pneumoniae*	99	Cereal baby food

*K. pneumoniae*=*Klebsiella pneumonia*

*K. pneumoniae* was not recovered from skim milk powder but was isolated from full cream milk powder 2 (20 %). *K. pneumoniae* was detected in powder infant formula (PIF), cereal baby food, and growing-up formula (fortified milk powder) as 1 (2.7%), 1 (6.25%), and 1 (5.5%), respectively, in baby food samples. However, no *K. pneumoniae* was observed in ready-to-feed baby milk and cereal baby food samples. No *K. pneumoniae* was detected in the ice cream and yogurt samples.

In addition, there was no zone of inhibition against amoxicillin, ampicillin, bacitracin, penicillin, lincomycin, clindamycin, and cloxacillin. However, these isolates were sensitive to levofloxacin, ciprofloxacin, amikacin, gentamycin, ertapenem, and chloramphenicol. All isolates were resistant to erythromycin, except for one intermediate isolate. In addition, some of these isolates exhibited different degrees of susceptibility to antibiotics such as kanamycin, tobramycin, nitrofurantoin, streptomycin, tetracycline, oxytetracycline, piperacillin cefoperazone/salbactum, ceftriaxone, cefoxitin, sulfamethoxazole/trimethoprim, ceftazidime, and cefotaxime. In addition, only one isolate was sensitive to methicillin, whereas the other isolates were resistant. In addition, all isolates were sensitive to aztreonam except for one isolate, which appeared intermediate. In addition, all isolates were sensitive to doxycycline and imipenem, except for two isolates that were resistant to these drugs. In addition, all isolates were sensitive to amoxicillin/clavulanic acid, except three isolates that were intermediate (Tables-3 and 4).

**Table 3:** Antibiogram of *Klebsiella pneumoniae* isolates.

No.	Antibiotics	1	2	3	4	5	6	7	8	9	10	11	12
1	Amoxycillin (10 μg)	R	R	R	R	R	R	R	R	R	R	R	R
2	Ampicillin (10 μg)	R	R	R	R	R	R	R	R	R	R	R	R
3	Bacitracin (10 μg)	R	R	R	R	R	R	R	R	R	R	R	R
4	Penicillin G (10 μg)	R	R	R	R	R	R	R	R	R	R	R	R
5	Methicillin (5 μg)	R	R	R	R	R	S	R	R	R	R	R	R
6	Erythromycin (15 μg)	R	R	R	I	R	R	R	R	R	R	R	R
7	Kanamycin (30 μg)	I	S	I	R	R	S	I	I	S	S	I	I
8	Lincomycin (10 μg)	R	R	R	R	R	R	R	R	R	R	R	R
9	Tobramycin (10 μg)	I	I	R	I	R	S	I	R	S	I	R	I
10	Levofloxacin (5 μg)	S	S	S	S	S	S	S	S	S	S	S	S
11	Clindamycin (2 μg)	R	R	R	R	R	R	R	R	R	R	R	R
12	Doxycycline (30 μg)	S	R	S	S	S	S	S	S	S	R	S	S
13	Cloxacillin (5 μg)	R	R	R	R	R	R	R	R	R	R	R	R
14	Nitrofurantoin (300 μg)	S	S	S	S	I	S	R	I	I	I	I	R
15	Oxytetracycline (30 μg)	I	R	R	I	I	I	R	R	I	R	R	I
16	Streptomycin (10 μg)	I	I	S	S	R	S	I	I	S	R	I	R
17	Tetracycline (30 μg)	S	S	S	I	S	S	S	S	S	R	S	I
18	Chloramphenicol (30 μg)	S	S	S	S	S	S	S	S	S	S	S	S
19	Sulphamethoxazole/trimethoprim (25 μg)	S	I	R	S	S	S	S	S	S	R	S	S
20	Amoxycillin/clavulanic acid (30 μg)	S	I	S	I	S	S	S	S	S	I	S	S
21	Piperacillin/tazobactum (100/10 μg)	S	I	I	S	S	S	I	S	S	S	I	I
22	Cephoxitin (30 μg)	I	R	I	I	S	I	R	I	S	R	I	R
23	Cefoperazone/salbactum (75/30 μg)	S	I	I	S	S	S	I	S	S	I	I	S
24	Cefotaxime (30 μg)	S	R	R	I	S	S	I	S	I	R	S	I
25	Ceftriaxone (30 μg)	S	I	I	S	S	S	I	S	S	S	S	I
26	Ceftazidim (30 μg)	R	R	R	R	I	R	R	R	R	R	R	R
27	Aztreonam (30 μg)	S	S	S	S	S	S	S	S	S	-	S	I
28	Imipenem (10 μg)	S	R	R	S	S	S	S	S	S	S	S	S
29	Ertapenem (10 μg)	S	S	S	S	S	S	S	S	S	S	S	S
30	Amikacin (30 μg)	S	S	S	S	S	S	S	S	S	S	S	S
31	Gentamycin (10 μg)	S	S	S	S	S	S	S	S	S	S	S	S
32	Ciprofloxacin (5 μg)	S	S	S	S	S	S	S	R	S	S	S	S
	IEH (10/750 μg)	-	MBL	MBL	-	-	-	-	-	-	-	-	-
Sensitivity %		51.5	27.2	36.3	45.4	51.5	63.6	36.3	45.4	57.5	30.3	42.4	33.3
Intermediate %		15.1	21.2	15.1	21.2	9	6	21.2	12.1	9	12.1	18.1	24.2
Resistance %		33.3	48.8	48.4	33.3	39.3	30.3	42.4	42.4	33.3	54.5	39.3	42.4

R: Resistant; I: Intermediate; S: Susceptible; MBL: Metallo-beta-lactamase.

**Table 4:** The resistance, intermediate, and sensitivity percentage of *K. pneumonia* isolates.

No.	Antibiotics	Resistance No. (%)	Intermediate No. (%)	Sensitive No. (%)
1	Amoxycillin (10 μg)	12 (100)	0 (0)	0 (0)
2	Ampicillin (10 μg)	12 (100)	0 (0)	0 (0)
3	Bacitracin (10 μg)	12 (100)	0 (0)	0 (0)
4	Penicillin G (10 μg)	12 (100)	0 (0)	0 (0)
5	Methicillin (5 μg)	11 (91.7)	0 (0)	1 (8.3)
6	Erythromycin (15 μg)	11 (91.7)	1 (8.3)	0 (0)
7	Kanamycin (30 μg)	2 (16.7)	6 (50)	4 (33.3)
8	Lincomycin (10 μg)	12 (100)	0 (0)	0 (0)
9	Tobramycin (10 μg)	4 (33.3)	6 (50)	2 (16.7)
10	Levofloxacin (5 μg)	0 (0)	0 (0)	12 (100)
11	Clindamycin (2 μg)	12 (100)	0 (0)	0 (0)
12	Doxycycline (30 μg)	2 (16.7)	0 (0)	10 (83.3)
13	Cloxacillin (5 μg)	12 (100)	0 (0)	0 (0)
14	Nitrofurantoin (300 μg)	2 (16.6)	5 (41.7)	5 (41.7)
15	Oxytetracycline (30 μg)	6 (50)	6 (50)	0 (0)
16	Streptomycin (10 μg)	3 (25)	5 (41.7)	4 (33.3)
17	Tetracycline (30 μg)	1 (8.3)	2 (16.7)	9 (75)
18	Chloramphenicol (30 μg)	0 (0)	0 (0)	12 (100)
19	Sulphamethoxazole/trimethoprim (25 μg)	2 (16.7)	1 (8.3)	9 (75)
20	Amoxycillin/clavulanic acid (30 μg)	0 (0)	3 (25)	9 (75)
21	Piperacillin/tazobactum (100/10 μg)	0 (0)	5 (41.6)	7 (58.3)
22	Cephoxitin (30 μg)	4 (33.3)	6 (50)	2 (16.7)
23	Cefoperazone+salbactum (75/30 μg)	0 (0)	5/(41.7)	7 (58.3)
24	Cefotaxime (30 μg)	3 (25)	4 (33.3)	5 (41.7)
25	Ceftriaxone (30 μg)	0 (0)	4 (33.3)	8 (66.7)
26	Ceftazidim (30 μg)	11 (91.7)	1 (8.3)	0 (0)
27	Aztreonam (30 μg)	0 (0)	1 (9.1)	10 (90.9)
28	Imipenem (10 μg)	2 (16.7)	0 (0)	10 (83.3)
29	Ertapenem (10 μg)	0 (0)	0 (0)	12 (100)
30	Amikacin (30 μg)	0 (0)	0 (0)	12 (100)
31	Gentamycin (10 μg)	0 (0)	0 (0)	12 (100)
32	Ciprofloxacin (5 μg)	1 (8.3)	0 (0)	11 (91.7)

Tables-5 and 6 show ARI index and MDR of all isolates against the 32 antibiotics used in this study.

**Table 5:** ARI and MDR of *K. pneumoniae* isolates.

No.	Antibiotics	Resistance isolates	ARI	MDR
1	Amoxycillin (10 μg)	12/100	0.03	36.3
2	Ampicillin (10 μg)	12/100	0.03	36.3
3	Bacitracin (10 μg)	12/100	0.03	36.3
4	Penicillin G (10 μg)	12/100	0.03	36.3
5	Methicillin (5 μg)	11/91.6	0.02	33.3
6	Erythromycin (15 μg)	11/91.6	0.02	33.3
7	Kanamycin (30 μg)	2/16.6	0.005	6
8	Lincomycin (10 μg)	12/100	0.03	36.3
9	Tobramycin (10 μg)	4/33.3	0.01	12.1
10	Levofloxacin (5 μg)	0/0	0	0
11	Clindamycin (2 μg)	12/100	0.03	36.3
12	Doxycycline (30 μg)	2/16.6	0.005	6
13	Cloxacillin (5 μg)	12/100	0.03	36.3
14	Nitrofurantoin (300 μg)	2/16.6	0.005	6
15	Oxytetracycline (30 μg)	6/50	0.01	18.1
16	Streptomycin (10 μg)	3/25	0.007	9
17	Tetracycline (30 μg)	1/8.3	0.002	3
18	Chloramphenicol (30 μg)	0/0	0	0
19	Sulphamethoxazole/trimethoprim (25 μg)	2/16.6	0.005	6
20	Amoxycillin/clavulanic acid (30 μg)	0/0	0	0
21	Piperacillin/tazobactum (100/10 μg)	0/0	0	0
22	Cephoxitin (30 μg)	4/33.3	0.01	12.1
23	Cefoperazone+salbactum (75/30 μg)	0/0	0	0
24	Cefotaxime (30 μg)	3/25	0.007	9
25	Ceftriaxone (30 μg)	0/0	0	0
26	Ceftazidim (30 μg)	11/91.6	0.02	33.3
27	Aztreonam (30 μg)	0/0	0	0
28	Imipenem (10 μg)	2/16.6	0.005	6
29	Ertapenem (10 μg)	0/0	0	0
30	Amikacin (30 μg)	0/0	0	0
31	Gentamycin (10 μg)	0/0	0	0
32	Ciprofloxacin (5 μg)	1/8.3	0.002	3

MDR=Multidrug resistance, ARI=Antibiotic resistance index, *K. pneumoniae*=*Klebsiella pneumonia*

**Table 6:** MARI of *K. pneumoniae*.

No. of isolates	No. of resistant antibiotics (Total)	MARI
1	11 (32)	0.33
2	16 (32)	0.48
3	16 (32)	0.48
4	11 (32)	0.33
5	13 (32)	0.39
6	10 (32)	0.3
7	14 (32)	0.42
8	14 (32)	0.42
9	11 (32)	0.33
10	18 (32)	0.54
11	13 (32)	0.39
12	14 (32)	0.42

MARI=Multi antibiotic resistance index, *K. pneumoniae*=*Klebsiella pneumoniae*

## Discussion

*K. pneumoniae* is commonly associated with nosocomial infections worldwide with high morbidity and mortality in humans and animals. The gastrointestinal tract and hands of the hospital staff are recognized as sources of *Klebsiella* contamination. *K. pneumoniae* was identified as a nosocomial pathogen responsible for a large-scale outbreak that occurred throughout the hospital environment and the food supply chain [20]. AMR is a new global healthcare crisis that has a significant impact on human health and the economy. This crisis is intensified by a lack of new antibiotics, particularly against Gram-negative pathogens, and the proliferation of high-risk MDR clones [21].

*K. pneumoniae* was recovered from three samples (6.5%) of raw cow’s milk (Table-1). This finding was slightly similar to that obtained by Tartor *et al*. [9] (7.2%) and Gaffer *et al*. [22] (8%). For instance, our results were less than those reported by Chaudhry *et al*. [1] (10.52%), Osman *et al*. [23] (9.6%), Garedew *et al*. [24] (16.7%), and Jayarao and Wang [25] (9.5%). Interestingly, Nobrega *et al*. [26] detected *K. pneumoniae* in most samples of milk from 25 lactating cows, indicating that raw milk may pose a risk of contamination by pathogenic bacteria. The results also highlight the importance of monitoring the quality and safety of raw milk and biological control. *K. pneumoniae* was detected in only one sample (20%) of she-camel’s milk. This finding was similar to that reported by Njage *et al*. [27] (33%). Interestingly, this is the first report to describe the isolation and identification of *K. pneumoniae* from camel milk in Libya.

The presence of *K. pneumoniae* in raw milk originates from the environment of dairy herds, and dairy herd mastitis may be due to the lack of hygiene in some dairy farms in Libya. According to Garedew *et al*. [24], the presence of *K. pneumoniae* in raw milk at various important critical points is associated with pre-milking udder preparation, milking treatment, and milking and storage equipment. Among all important control points, the most critical risk factors were poor hygiene practices during the transport of containers at milk collection points and processing plants. However, *K. pneumoniae* was not detected in eight samples of UHT milk. This may be due to heat treatment during production.

*K. pneumoniae* was recovered from 4 samples (19%) of Maasora cheese, 1 sample (7.6%) of Ricotta cheese, and 1 sample (16.6%) of imported soft cheese (Table-1). These results are similar to those reported by Gaffer *et al*. [22], who found *K. pneumoniae* in Damietta cheese (12%) and Kariesh cheese (8%). Massa *et al*. [28] found that 90% of mozzarella cheese samples were contaminated by *K. pneumoniae*. Remarkably, Massa *et al*. [28] reported that *K. pneumoniae* is one of the most common pathogens that cause cheese spoilage. In addition, in Libya, the majority of workers handling locally produced dairy products, such as milk and cheese, do not comply with the proper hygiene guidelines for preparation.

*K. pneumoniae* was recovered from one sample of raw fermented milk (3.5%) (Table-3); however, these findings are not consistent with those obtained by Abushaala and Alwoshesh [29] (12.9%) and Njage *et al*. [27] (33%), who found *K. pneumoniae* in natural fermented milk. On the other hand, no *K. pneumoniae* was found in the yogurt samples (0%), which may be because *K. pneumoniae* could not tolerate the high acidity in this product.

In the present study, *K. pneumoniae* was isolated from one sample of PIF (2.7%), cereal baby food 1 (6.25%), and growing-up formula (fortified milk) 1 (5.5%). These results were nearly similar to those reported by Yao *et al*. [30] (4.3%), Sani and Lim [31] (6.6%, (7.14%), Oonaka *et al*. [32] (4.6%), and Estuningsih *et al*. [33] (4%) (Table-1). However, the results of our study are not consistent with those obtained by Zhou *et al*. [34] (26.7%), Sani and Lim [31] (6.6%), and Muytjens *et al*. [35] (9.2%), respectively.

Baby milk powder may contain *K. pneumoniae* due to the addition of heat-sensitive ingredients such as vitamins, minerals, dried rice, and fruits. In addition, the microbiological safety of PIF has recently received considerable attention. This was mainly due to neonatal infections caused by Enterobacteriaceae, including *Cronobacter sakazakii* and *K. pneumoniae*, associated with PIF contamination. These products are not sterile but are expected to comply with international microbiological standards [34].

*K. pneumoniae* is another opportunistic bacterial pathogen present in PIF, as reported by Feng *et al*. [36]. The present study demonstrated that the presence of *K. pneumoniae* in PIF is considered to be a major cause of infections among infants resulting from the improper preparation and conservation of these products, especially at hospitals and homes, which could cause a health risk for infants. *Klebsiella* species have been associated with necrotizing enterocolitis in infants, as documented by Coleman *et al*. [37]. These species have been classified as category B organisms and are intended to cause infections in infants. As reported by Liu *et al*. [20], *K. pneumoniae* has been recovered from modular industrialized enteral diets and milk-based and food supplements used in public hospitals. As documented by Wareth *et al*. [14], *K. pneumoniae* is among the most frequently isolated bacterial species in milk substitution formulas for infants collected from 35 countries. Pasteurized milk and whole milk powder samples collected from retail shops in Mexico, there has been a high incidence of MDR strains, which represents a public health hazard.

In the present study, *K. pneumoniae* was not recovered from ice cream samples, which may have been due to the inability of *K. pneumoniae* to survive in freezing temperatures. However, Gaffer *et al*. [22] detected only one isolate (4%) of *K. pneumoniae* in ice cream samples (Table-1).

*K. pneumoniae* was isolated from 2 samples (20%) of full cream milk powder (Table-1). For instance, Wareth *et al*. [14] obtained 24 isolates of *K. pneumoniae* from milk powder producer companies in Libya. To the best of our knowledge, this is the first report to describe the presence of *K. pneumoniae* in milk powder in Libya, which may be a result of post-processing contamination. However, no *K. pneumoniae* was found in 6 (0%) cases of skim milk powder.

Recently, *K. pneumoniae* has been detected in human samples, again emphasizing the concept of transmission between animal/animal products, including milk, from/to humans and animals during the last decade. MDR *K. pneumoniae* isolates have also been widely detected in milk samples [9]. Moreover, *K. pneumoniae* is considered a major vehicle of resistance genes from environmental sources to clinically important bacteria and some isolates can carry acquired AMR genes or plasmids between the environment, humans, and animals [12]. In this study, two isolates were MBL producers, as shown in Table-3.

Liang *et al*. [6] isolated *K. pneumoniae* from different sources, including humans, the environment, and animals, supporting direct/indirect transmission between humans and animals, in accordance with the recent global concept of the dissemination of mobilized colistin resistance genes from animals to humans. This result emphasizes the concept of “One Health”: Human health is connected to the health of animals and the environment.

## Conclusion

*K. pneumoniae* is now recognized as an urgent threat to human health because of the emergence of multidrug-resistant strains associated with hospital outbreaks and hypervirulent strains associated with severe community-acquired infections. *K. pneumoniae* is ubiquitous in the environment and can colonize and infect both plants and animals. This study emphasizes the relationship between *K. pneumoniae* and raw milk, cheese, milk powder, and infant milk retailed in Libya, which is considered a risk for human health because many of these products do not comply with international microbiological criteria and/or Libyan standards. There is a need to take the necessary measures to ensure effective hygiene practices during production in dairy factories, handling, and distribution on the market, in particular at a locally small production scale. Moreover, our results showed that high-level MDR was present among most isolates against different antibiotics. Interestingly, this is the first study to document the transmission of MDR *K. pneumoniae* through milk and milk products, which constitutes a huge threat to food safety, especially to the public health of adults and neonates.

## Data Availability

The supplementary data can be available from the corresponding author upon a reasonable request.
